# Factors Associated with Neuroimaging Abnormalities in Children with Afebrile Seizure: A Retrospective Multicenter Study

**DOI:** 10.5811/westjem.2022.12.57505

**Published:** 2023-02-27

**Authors:** Seungho Woo, Sangun Nah, Minsol Kim, Sangil Kim, Dongwook Lee, Jaewook Lee, Jieun Moon, Sangsoo Han

**Affiliations:** * Soonchunhyang University Bucheon Hospital, Department of Emergency Medicine, Bucheon, Republic of Korea; † Soonchunhyang University Bucheon Hospital, Department of Pediatrics, Bucheon, Republic of Korea; ‡ Soonchunhyang University Seoul Hospital, Department of Emergency Medicine, Seoul, Republic of Korea; § Soonchunhyang University Cheonan Hospital, Department of Emergency Medicine, Cheonan, Republic of Korea; || Soonchunhyang University Bucheon Hospital, Department of Radiology, Bucheon, Republic of Korea; # Soonchunhyang University Bucheon Hospital, Clinical Trial Center, Department of Biostatistics, Bucheon, Republic of Korea

## Abstract

**Introduction:**

Neuroimaging is recommended for patients with seizures to identify intracranial pathology. However, emergency physicians should consider the risks and benefits of neuroimaging in pediatric patients because of their need for sedation and greater sensitivity to radiation than adults. The purpose of this study was to identify associated factors of neuroimaging abnormalities in pediatric patients experiencing their first afebrile seizure.

**Methods:**

This was a retrospective, multicenter study that included children who presented to the emergency departments (ED) of three hospitals due to afebrile seizures between January 2018–December 2020. We excluded children with a history of seizure or acute trauma and those with incomplete medical records. A single protocol was followed in the three EDs for all pediatric patients experiencing their first afebrile seizure. We performed multivariable logistic regression analysis to identify factors associated with neuroimaging abnormalities.

**Results:**

In total, 323 pediatric patients fulfilled the study criteria, and neuroimaging abnormalities were observed in 95 patients (29.4%). Multivariable logistic regression analysis showed that Todd’s paralysis (odds ratio [OR] 3.72, 95% confidence interval [CI] 1.03–13.36; P=0.04), absence of poor oral intake (POI) (OR 0.21, 95% CI 0.05–0.98; P=0.05), lactic acidosis (OR 1.16, 95% CI 1.04–1.30; P=0.01), and higher level of bilirubin (OR 3.33, 95% CI 1.11–9.95; P=0.03) were significantly associated with neuroimaging abnormalities. Based on these results, we constructed a nomogram to predict the probability of brain imaging abnormalities.

**Conclusion:**

Todd’s paralysis, absence of POI, and higher levels of lactic acid and bilirubin were associated factors of neuroimaging abnormalities in pediatric patients with afebrile seizure.

## INTRODUCTION

Seizures are one of the most common neurological disorders in children, with a prevalence of approximately 1%.^1^,^2^ Febrile seizure is the most common type of pediatric seizure.^3^ In comparison, afebrile seizure is relatively uncommon, but it is clinically significant. Numerous studies have reported a higher risk of abnormal neuroimaging findings and recurrence of seizure.^4^–^6^

Previous studies have recommended electroencephalography (EEG) and/or neuroimaging after the first afebrile seizure.^7^–^12^ However, pediatric seizure patients rarely undergo EEG in the emergency department (ED) due to differences in staffing and problems such as limited EEG lab availability at night.^13^ In contrast, brain imaging tests, including computed tomography and magnetic resonance imaging (MRI), are relatively accessible in the ED. In a previous multicenter study, a majority of the seizure patients (81%) underwent neuroimaging studies in the ED, while EEG was performed in only 3% of the patients at the same time due to lack of testing availability.^14^ Furthermore, emergent brain imaging allows clinicians to identify intracranial pathologies and the need for immediate intervention in children with afebrile seizures.^9^–^12^

Clinicians should carefully consider the risks and/or benefits of brain imaging in pediatric patients because they typically require sedation and are much more sensitive to ionizing radiation than adults.^15^,^16^ Therefore, it is important to identify associated factors of neuroimaging abnormalities in pediatric patients with first afebrile seizure. Clinical guidelines for evaluating the first afebrile seizure in children, which were published by the American Academy of Neurology, Child Neurology Society, and American Epilepsy Society, suggest that emergent neuroimaging should be performed in all pediatric patients who present with Todd’s paralysis or have not returned to baseline status within a few hours after the seizure.^17^ However, previous studies on emergent neuroimaging in pediatric patients with afebrile seizure did not take lab results into consideration.

The purpose of this study was to identify associated factors of abnormalities on emergent neuroimaging tests after the first afebrile seizure episode in children based on historical findings, physical examination, and lab results.

## METHODS

### Study Design and Setting

This retrospective, multicenter study recruited pediatric patients who presented to three university hospitals (in Seoul, Chungcheong, and Gyeonggi, Republic of Korea) with seizures between January 2018–December 2020. These EDs serve approximately 40,000, 50,000, and 60,000 patients per year, respectively. Children aged 1 month to 18 years, who were afebrile for at least 24 hours and presented with their first seizure, were enrolled. We excluded children with a known seizure disorder, acute trauma, or incomplete electronic health records (EHR). The study was approved by our hospital institutional review board (IRB file no. 2021-03-030).

**Population Health Research Capsule**

What do we already know about this issue?

*Neuroimaging is recommended in children presenting with a first afebrile seizure in the emergency department. However, there are risks of sedation and radiation*.

What was the research question?

*What factors are associated with neuroimaging abnormalities in children with a first afebrile seizure?*

What was the major finding of the study?

*Abnormal neuroimaging findings were present in 29% of patients. Higher levels of lactate and bilirubin, Todd’s paralysis, and the absence of poor oral intake were factors associated with neuroimaging abnormalities*.

How does this improve population health?

*Our model could help emergency physicians identify pediatric patients who require brain imaging based on laboratory results and clinical findings*.

### Patient Identification and Data Collection

We reviewed the EHR of 2,009 patients evaluated at the three EDs for seizures between January 2018–December 2020. In our retrospective study, we estimated a sample size based on a previous study about calculating adequate sample size for developing a clinical prediction model.^18^ Data was extracted by three experienced reviewers. In case of discrepancy, the data extracted by the most senior reviewer was recorded. The following data was extracted from the medical records: age; gender; symptoms (ie, headache, vomiting, and poor oral intake [POI]); past history (related to birth, neonatal intensive care unit [NICU] admission, and family); number of seizures in the first 24 hours after presentation; duration of seizure; presence of Todd’s paralysis; postictal features; seizure type; physical exam findings (ie, neck stiffness and Babinski sign); laboratory results (ie, blood pH, complete blood cell count, and bicarbonate, lactic acid, blood urea nitrogen, creatinine, glucose, albumin, bilirubin, aspartate aminotransferase, alanine transaminase, and C-reactive protein [CRP] levels). POI was defined as a lack of interest in feeding or a problem receiving the proper amount of nutrition and Todd’s paralysis was defined as a neurological condition, in which a seizure is followed by a brief period of temporary paralysis. Also, lactate level above 2.0 mg/dL and bilirubin level above 1.2 mg/dL were considered abnormal in our laboratory results. Data collection was conducted in accordance with the recommendations of Worster et. al to reduce bias and comply with standards for EHR review.^19^

### Neuroimaging Studies

Because the three study EDs share the same medical center, a single protocol was followed for all pediatric patients experiencing their first afebrile seizure. Therefore, most of the patients underwent brain imaging tests. We excluded patients for whom brain imaging was performed 24 hours post seizure, characterizing them as having incomplete EHR. A single neuroradiologist reviewed the neuroimaging studies. The neuroimaging findings were classified as cyst (ie, neuroglial or arachnoid cyst), infarction, hemorrhage, mass, encephalitis, dysplasia (ie, developmental venous anomaly or lissencephaly), focal (ie, focal heterotopia or focal encephalomalacia), cortical edema, periventricular leukomalacia [PVL], and non-specific lesions (ie, non-specific, increased T2 signal intensity in periventricular white matter).^10^ In brain imaging studies, 157 patients (48.6%) underwent brain CT, 268 patients (83.0%) underwent MRI, and 102 patients (31.6%) underwent both CT and MRI.

### Statistical Analysis

We present data as absolute numbers or relative frequencies for categorical variables, and as medians with interquartile ranges for continuous variables. *P*-values < 0.05 were considered to indicate statistical significance. We used Fisher’s exact test or the chi-squared test to analyze categorical variables and the Mann-Whitney U test to analyze continuous variables. The Shapiro-Wilk test showed that all continuous variables did not follow a normal distribution. Multivariable logistic regression analysis was performed on factors that were statistically significant in the univariable logistic regression analysis and variables reported to be significantly associated in previous studies.^11^,^12^,^17^ We calculated odds ratios (OR) with 95% confidence intervals (CI) to quantify the associations between the various factors and neuroimaging abnormalities. A nomogram based on the multivariable logistic analysis was constructed to predict brain imaging abnormalities. We performed statistical analyses using SPSS version 26.0 (IBM Corp., Armonk, NY) and R version 4.1.3 (R Foundation for Statistical Computing, Vienna, Austria).

## RESULTS

**Figure 1:**
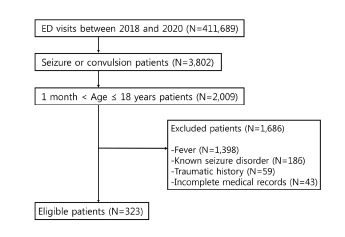
Flow chart of patient selection for study of neuroimaging in first afebrile pediatric seizure. *ED*, emergency department.

A total of 411,689 patients visited the three EDs during the study period, including 2,009 pediatric patients who visited the EDs due to a seizure. We excluded patients with fever (1,398), history of seizure disorder (186), history of acute trauma (59), and incomplete EHR including absence of neuroimaging tests (43). Finally, 323 patients were enrolled in the study (Figure 1).

### General Characteristics

Table 1 shows the baseline characteristics of the patients. The median age was 7 (2.5–11.5) years. In total, 26 patients (8.05%) were admitted to the NICU, and 48 (14.86%) had a familial history of seizures. Headache and POI were present in 33 (10.22%) and 24 (7.43%) patients, respectively. The median number of seizures was 1 (1–1) and the median seizure duration was 3 (2–5) minutes. Twelve patients (3.72%) developed Todd’s paralysis, and 231 (71.52%) developed a postictal state. Most seizures were of the generalized tonic-clonic (GTC) type (277, 85.76%), followed by focal type (43, 13.31%) and secondary GTC type (3, 0.93%).

### Comparison of the Two Groups with Normal and Abnormal Neuroimaging

The differences between the normal and abnormal neuroimaging groups are summarized in Table 2. Abnormal neuroimaging findings were present in 95 patients (29.41%). No significant differences were observed in age, gender, medical history, number of seizures, or seizure type between the two groups. On physical examination, the number of patients with neck stiffness was greater in the abnormal neuroimaging group but showed no significant difference (3.16% and 0.44%, respectively; *P* = 0.08), and Babinski sign was identified in only one case, making it difficult to have a significant difference (1.05% and 0%, respectively; *P* = 0.29).

Fewer patients in the abnormal neuroimaging group had POI compared to the normal neuroimaging group (2.11% and 9.65%, respectively; *P*=0.02). In addition, Todd’s paralysis was observed more frequently in the abnormal than normal neuroimaging group (7.37% and 2.19%, respectively; *P*=0.05). Compared to the normal neuroimaging group, patients in the abnormal neuroimaging group had higher levels of lactic acid (2 and 2.2 milligrams per deciliter [mg/dL], respectively; *P*=0.02) and bilirubin (0.34 and 0.4 mg/dL, respectively; *P*=0.03), and lower levels of albumin (4.6 and 4.5 grams/dL, respectively; *P*=0.02) and CRP (1.3 and 0.7 mg/L, respectively; *P*=0.03).

### Main Outcomes

The results of the univariable and multivariable logistic regression analyses are shown in Table 3. Neck stiffness did not show a significant difference in univariable logistic regression analysis (OR 7.402, 95% CI 0.760–72.087; *P*=0.09). Todd’s paralysis was significantly associated with abnormal neuroimaging findings (OR 3.718, 95% CI 1.034–13.364; *P*=0.04). Furthermore, POI was inversely associated with neuroimaging abnormalities (OR 0.213; 95% CI 0.046–0.976; *P*=0.05). The lactic acid (OR 1.161, 95% CI 1.035–1.302; *P*=0.01) and bilirubin (OR 3.330, 95% CI 1.114–9.952; *P*=0.03) levels were significantly associated with abnormal neuroimaging findings.

**Figure 2:**
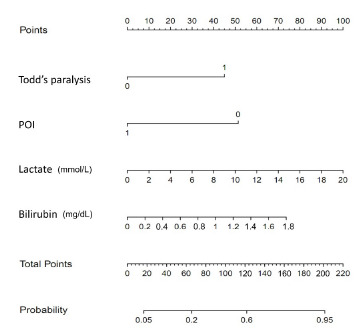
Nomogram for predicting neuroimaging abnormalities in pediatric patients experiencing their first afebrile seizure. Each factor was rated on the top visible point scale (0–100), and the probability of neuroimaging abnormalities was predicted by summing the scores of all factors. For example, a patient with Todd’s paralysis and a bilirubin level of 1.0 mg/dL would have 45 and 40 points for each factor. After summing the scores, total points are 85, and the probability of neuroimaging abnormalities would be 0.4 for the patient. *POI*, poor oral intake.

We used these factors to construct a nomogram (Figure 2) for predicting the probability of abnormal neuroimaging findings. The nomogram showed that lactate was the strongest associated factor of neuroimaging abnormalities, followed by bilirubin, absence of POI, and Todd’s paralysis. Each associated factor was rated on a point scale (0–100). The probability of neuroimaging abnormalities was predicted by summing the scores of all factors. For example, a patient with Todd’s paralysis and a bilirubin level of 1.0 mg/dL would have 45 and 40 points for each factor. After summing the scores, there were 85 total points, and the probability of neuroimaging abnormalities was 0.4 for the patient.

### Neuroimaging Studies

**Figure 3:**
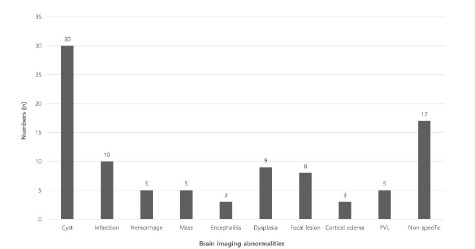
Classification of neuroimaging abnormalities. *PVL*, periventricular leukomalacia.

In total, 95 patients had abnormal neuroimaging findings, which are summarized in Figure 3. The most common finding was cyst (30, 31.58%), followed by non-specific findings (19, 17.89%), infarction (10, 10.53%), dysplasia (9, 9.47%), and focal lesion (8, 8.42%). Hemorrhage (5, 5.26%), mass (5, 5.26%), PVL (5, 5.26%), encephalitis (3, 3.16%), and cortical edema (3, 3.16%) were also frequently identified.

## DISCUSSION

In this multicenter retrospective study, we investigated associated factors of emergent neuroimaging abnormality in children with first-onset, non-febrile seizure from historical findings, physical exam, and lab results. Todd’s paralysis, absence of POI, and higher levels of lactate and bilirubin were significantly associated with the abnormalities of brain imaging tests. In a previous prospective observational study, Paramasivam et al reported that 22 of 65 patients (33.85%) experiencing new-onset afebrile seizures had abnormal neuroimaging findings.^20^ In addition, Al-Shami et al reported that 32 of 96 pediatric patients (33.33%) with afebrile seizures had neuroimaging abnormalities that were considered to be clinically significant by an experienced neuroradiologist.^12^ Similarly, we found that 95 pediatric patients (29.41%) with afebrile seizures had abnormal neuroimaging findings.

Current practice guidelines for the evaluation of children experiencing their first afebrile seizure suggest that manifestations of focal seizures, such as Todd’s paralysis and persistent mental status change, are risk factors for neuroimaging abnormalities.^17^ Aprahamian et al reported that Todd’s paralysis and age <18 months were associated factors of neuroimaging abnormalities requiring urgent care.^11^ Amagasa et al also reported that neurological disorder, including impaired awareness, Todd’s paralysis, and ataxia in physical examinations, was a risk factor for brain imaging abnormalities in children with first afebrile seizure.^21^ In our study, we investigated both Todd’s paralysis and physical findings such as neck stiffness and Babinski sign. We found that Todd’s paralysis was a significant associated factor of neuroimaging abnormalities, while age, neck stiffness, and Babinski sign were not. Whether age is related to neuroimaging abnormalities varies from study to study.^11^,^20^ Similar to our study, Paramasivam et al reported no significant association between age and gender with neuroimaging abnormalities.^20^ In our study, neck stiffness and Babinski sign showed higher numbers in the abnormal neuroimaging group. However, the number of patients with neck stiffness or Babinski sign was too small to determine the association. Further research on more detailed neurological examinations in pediatric afebrile seizure patients is needed.

In pediatric patients, POI may indicate several metabolic abnormalities such as hypoglycemia, hyponatremia, and hypocalcemia that can cause convulsions.^22^,^23^ According to a previous study, 95.2% of infants and young children with gastroenteritis complained of POI and presented with ketosis or hypoglycemia, as well as hyponatremic dehydration.^24^ In addition, pediatric patients with certain metabolic disorders, such as glucose transporter type 1 deficiency syndrome, may experience seizures; however, the syndrome is rare with an estimated birth incidence of 1 in 24,300 according to a previous study. For these patients, a ketogenic diet may prevent seizures.^24^–^26^ In our study, absence of POI was an associated factor of neuroimaging abnormalities, where absence of POI may indicate intracranial pathology rather than metabolic problems in children.

With regard to lab results, previous studies reported that the lactic acid level is an excellent biomarker for discriminating seizures from psychogenic episodes and syncope, although the underlying mechanism is unclear.^27^,^28^ Gunawan et al recently reported a correlation between serum lactate level and abnormal brain MRI findings in children with status epilepticus.^29^ Lactate elevation and adenosine triphosphate depletion during the early phase of seizures are related to hypermetabolic neuronal damage.^29^ Similarly, lactic acid was a strong associated factor of neuroimaging abnormalities in our pediatric patients experiencing their first afebrile seizure. In addition, Shapiro et al reported that unbound and free unconjugated bilirubin can selectively damage the central nervous system in the developing brain.^30^ However, bilirubin toxicity to the brain is only applicable to early neonatal age. In our study, a higher level of bilirubin was found to be significantly associated with neuroimaging abnormalities in pediatric patients with first afebrile seizure. Further research on how bilirubin affects the brain in pediatric afebrile seizure patients is needed.

The main strength of our study is that it presents a nomogram based on lab results and clinical findings, such as Todd’s paralysis and POI, to predict neuroimaging abnormalities in children experiencing their first afebrile seizure. Because this data can be easily acquired in the ED, our predictive model could help identify pediatric patients who require brain imaging. To the best of our knowledge, this is the first study to identify associated factors of neuroimaging abnormalities in patients experiencing afebrile seizure based on symptoms and lab findings.

## LIMITATIONS

Our study had several limitations. First, selection bias may have occurred due to the retrospective study design. However, the three study EDs shared the same protocol for pediatric afebrile seizure, which would minimize selection bias. While our goal was to investigate all known factors with regard to neuroimaging abnormalities in pediatric afebrile seizure patients, some may have been missed due to the limitations of a retrospective study design.^17^, ^20^ Second, our neuroimaging findings are unclear as to whether the findings were the cause or the consequence of seizures. Third, caution should be exercised when generalizing the results because of regional differences. However, the study hospitals are located in major population centers in the Republic of Korea (Seoul, Bucheon, and Cheonan), which should improve the generalizability. Finally, while we estimated a sample size considering a previous study, the sample size might be too small to be generalized.^18^ To overcome these limitations, large-scale prospective studies on neuroimaging abnormalities in pediatric patients with afebrile seizures are needed.

## CONCLUSION

We found that Todd’s paralysis, absence of POI, and higher levels of lactic acid and bilirubin were significant associated factors of neuroimaging abnormalities in pediatric patients with first afebrile seizure. We then constructed a nomogram to aid emergency physicians in making decisions quickly about initiating emergent neuroimaging tests for children with first afebrile seizure, as pediatric seizure patients with these findings should be closely monitored.

## Figures and Tables

**Table 1:** Baseline characteristics of pediatric patients with afebrile seizures.

	Total (N = 323)
Age at onset, years	7 [2.5–11.5]
Gender, n (%)	
Female	141 (43.65)
Male	182 (56.35)
Past history	
IUP, weeks	38 [37–39.3]
Birth weight, kilograms	3 [2.7–3.3]
C-sec, n (%)	147 (45.51)
NICU admission, n (%)	26 (8.05)
Familial history, n (%)	48 (14.86)
Symptoms, n (%)	
Headache	33 (10.22)
Vomiting	69 (21.36)
POI	24 (7.43)
Seizure features	
Seizure count, n	1 [1–1]
Duration, minutes	3 [2–5]
Todd’s paralysis, n (%)	12 (3.72)
Postictal state, n (%)	231 (71.52)
Type, n (%)	
GTC	277 (85.76)
Focal	43 (13.31)
Secondary GTC	3 (0.93)

Categorical variables are presented as numbers (percentage). Continuous variables are presented as medians [interquartile range].

*IUP*, intrauterine pregnancy; *C-sec*, caesarean section; *NICU*, neonatal intensive care unit; *POI*, poor oral intake; *GTC*, generalized tonic-clonic.

**Table 2:** Comparison of baseline characteristics between the two groups.

	Normal (n = 228)	Abnormal (n = 95)	P-value
Age at onset, years	6 [2–11]	7 [4–12]	0.39
Gender, n (%)			0.62*
Female	102 (44.74)	39 (41.05)	
Male	126 (55.26)	56 (58.95)	
Past history			
IUP, weeks	38 [37–39]	38 [37–40]	0.43
Birth weight, kilograms	3 [2.9–3.3]	3 [2.7–3.3]	0.70
C-sec, n (%)	100 (43.86)	47 (49.47)	0.39*
NICU admission, n (%)	15 (6.58)	11 (11.58)	0.18*
Familial history, n (%)	33 (14.47)	15 (15.79)	0.74*
Symptoms, n (%)			
Headache	21 (9.21)	12 (12.63)	0.42*
Vomiting	48 (21.05)	21 (22.11)	0.88*
POI	22 (9.65)	2 (2.11)	0.02*
Seizure features			
Seizure count, n	1 [1–1]	1 [1–1]	0.38
Duration, minutes	3 [1–5]	4 [2–7.5]	0.06
Todd’s paralysis, n (%)	5 (2.19)	7 (7.37)	0.05**
Postictal confusion, n (%)	156 (68.42)	75 (78.95)	0.06*
Type, n (%)			0.62**
GTC	198 (86.84)	79 (83.16)	
Focal	28 (12.28)	15 (15.79)	
Secondary GTC	2 (0.88)	1 (1.05)	
Physical examinations, n (%)			
Neck stiffness	1 (0.44)	3 (3.16)	0.08**
Babinski sign	0 (0)	1 (1.05)	0.29**
Laboratory results			
pH	7.35 [7.31–7.39]	7.34 [7.29–7.38]	0.36
Bicarbonate, mmol/L	23.8 [22.1–26.0]	23.5 [21.2–25.5]	0.83
Lactate, mg/dL	2 [1.4–3.0]	2.2 [1.6–3.8]	0.02
WBC, 10^3^/μL	8.59 [6.8–11.3]	8.79 [6.7–11.9]	0.69
Hb, g/dL	12.9 [12.1–13.6]	12.7 [12.1–13.7]	0.71
PLT, 10^3^/μL	294 [249.8–346.3]	294 [256.5–353.5]	0.89
BUN, mg/dL	11.7 [9.0–13.6]	11.2 [9.1–13.1]	0.28
Cr, mg/dL	0.5 [0.39–0.66]	0.5 [0.4–0.7]	0.11
Glucose, mg/dL	102 [92–115]	104 [94.5–127]	0.14
Albumin, g/dL	4.6 [4.4–4.8]	4.5 [4.2–4.7]	0.02
Bilirubin, mg/dL	0.34 [0.22–0.48]	0.4 [0.3–0.53]	0.03
AST, U/L	29 [22–38]	28 [22–35]	0.34
ALT, U/L	14 [11.0–20.3]	14 [12–19]	0.73
CRP, mg/L	1.3 [0.3–4.6]	0.7 [0.3–2.1]	0.03

Categorical variables are presented as numbers (percentage) and were analyzed using the *Pearson chi-squared test or **Fisher’s exact test. Continuous variables are presented as median [interquartile range] and were analyzed by Mann–Whitney U test. (All continuous variables did not have a normal distribution.)

*IUP*, intrauterine pregnancy; *C-sec*, caesarean section; *NICU*, neonatal intensive care unit; *POI*, poor oral intake; *GTC*, generalized tonic-clonic; *pH*, potential of hydrogen; *WBC*, white blood cell; *Hb*, hemoglobin; *PLT*, platelet; *BUN*, blood urea nitrogen; *Cr*, creatinine; *AST*, aspartate aminotransferase; *ALT*, alanine transaminase; *CRP*, C-reactive protein.

**Table 3:** Univariable and multivariable logistic regression analyses of risk factors for neuroimaging abnormalities in pediatric afebrile seizure patients.

	Univariable		Multivariable	
	OR (95% CI)	P-value	OR (95% CI)	P-value
Seizure features				
Duration	1.017(0.994–1.040)	0.16		
Postictal confusion	1.731(0.982–3.051)	0.06		
Todd’s paralysis	3.548(1.097–11.475)	0.03	3.718(1.034–13.364)	0.04
Symptoms				
POI			0.213(0.046–0.976)	0.05
Physical examinations				
Neck stiffness	7.402(0.760–72.087)	0.09		
Laboratory results				
CRP	0.899(0.767–1.052)	0.18		
Albumin	0.388(0.178–0.845)	0.02	0.531(0.218–1.289)	0.16
Bilirubin	3.078(1.167–8.122)	0.02	3.330(1.114–9.952)	0.03
Lactate	1.163(1.047–1.292)	0.01	1.161(1.035–1.302)	0.01

*OR*, odds ratio; *CI*, confidence interval; *POI*, poor oral intake; *CRP*, C-reactive protein.
